# Treatment of Venous Thromboembolism in Pediatric Patients

**DOI:** 10.3389/fped.2017.00026

**Published:** 2017-02-28

**Authors:** Lynn Malec, Guy Young

**Affiliations:** ^1^BloodCenter of Wisconsin, Milwaukee, WI, USA; ^2^Medical College of Wisconsin, Milwaukee, WI, USA; ^3^Department of Pediatrics, Division of Hematology, Children’s Hospital of Los Angeles, Los Angeles, CA, USA

**Keywords:** venous thrombosis, thromboembolism, pediatrics, treatment, anticoagulants

## Abstract

Given the increased incidence of venous thromboembolism (VTE) in pediatric patients, which has been associated with increased survival of medically complex patients and increased use of invasive supportive measures, it is important to understand treatment options and unique aspects of anticoagulant use in children. The objective of this mini-review is to outline the goals of treatment, treatment options, and adverse events associated with the use of anticoagulants in pediatric patients with VTE.

## Background

Venous thromboembolism (VTE), especially hospital-acquired VTE, is increasingly recognized in pediatric patients. The incidence of VTE in hospitalized children has increased approximately 70% over a 6-year period and is thought to affect approximately 1 in every 200 hospitalized children (1). The rise in VTE is largely attributed to increased use of invasive support of critically ill patients, especially with the use of central venous access devices, which can lead to line-related VTE, and the improved survival of patients with complex medical conditions. Recent efforts have been made to better understand aspects of VTE in this patient population including risk factors for development of thrombosis, therapeutic outcomes, risks for recurrence, and long-term prognosis as these may differ from those in adult patients.

When considering treatment options in children, it is important to consider ways in which use of anticoagulants in pediatric patients may differ from adults. As outlined in the American College of Chest Physicians CHEST Guidelines for “Antithrombotic Therapy in Neonates and Children,” some of these important differences include (1) “epidemiology of thromboembolism in pediatric patients differs from that seen in adults,” (2) “hemostatic system is a dynamic, evolving entity that likely affects not only the frequency and natural history of thromboembolism in children but also the response to therapeutic agents,” (3) “distribution, binding, and clearance of antithrombotic drugs are age dependent,” (4) “limited vascular access reduces the ability to effectively deliver some antithrombotic therapies and can influence the choice of antithrombotic agent,” (5) “specific pediatric formulations of antithrombotic drugs are not available, making accurate, reproducible dosing difficult,” and (6) “dietary differences make the use of oral vitamin k antagonists particularly difficult” (2). With these considerations in mind, this article focuses on therapeutic options for VTE in children, which are important in order to optimize care and outcomes in this cohort.

## Goals of Treatment

The goals of treatment of pediatric VTE overlap with those of adult patients. The initial goal of anticoagulation is to halt clot progression. With the initiation of parenteral or enteral anticoagulation, clot stabilization will typically occur, thus preventing a thrombus from expanding in length to involve additional venous segments, or expanding in circumferential diameter. Use of conventional anticoagulants will not cause clot breakdown, rather the body relies on its endogenous fibrinolytic system to dissolve the thrombus. Another important goal of treatment of VTE is the prevention of embolization of the thrombus from its original site to areas such as the lungs or central nervous system. When embolization does occur, it can substantially increase the morbidity and mortality associated with VTE (3).

With use of anticoagulation, an additional goal is prevention of VTE recurrence. The specific role of anticoagulation, including duration of therapy, is not clearly defined in regards to recurrence prevention. To date, no adequately powered pediatric study has addressed this issue; however, a current randomized controlled trial (RTC) is underway that has demonstrated feasibility in the initial pilot phase (4). The Duration of Therapy for Thrombosis in Children and Young Adults (Kids-DOTT) trial is a multicenter RTC investigating non-inferiority of a 6-week (shortened) versus 3-month (conventional) duration of anticoagulation in patients aged <21 years with provoked venous thrombosis with primary efficacy and safety endpoints of symptomatic recurrent VTE and anticoagulant-related bleeding.

In medically complex patients dependent on venous access for life sustaining measures, including those with congenital heart disease requiring repeated cardiac catheterization and short bowel syndrome requiring long-term parenteral nutrition, recurrent VTE that limits adequate venous access can become a life-limiting condition. In this setting, use of anticoagulants for secondary prophylaxis is often considered to reduce the risk of VTE recurrence. Data regarding efficacy of specific agents and complications in secondary prophylaxis in an RTC are largely lacking in pediatrics.

A potential debilitating long-term complication of VTE is the development of post-thrombotic syndrome (PTS). PTS arises as a result of chronic venous occlusion or valvular disruption leading to venous hypertension. Symptoms of PTS include limb heaviness, swelling, pain, cramping, and ulceration. Instituting anticoagulation early is crucial in order to minimize risk of clot propagation and to encourage clot resolution, both thought to reduce the risks of PTS in the pediatric patient population.

## Treament Options

The most common treatment options for VTE include unfractionated heparin (UFH), low molecular weight heparin (LMWH), and warfarin; other options include fondaparinux and the direct thrombin inhibitors (DTIs). This article will focus on the use of these parenteral and enteral anticoagulants; published data on the direct oral anticoagulants (DOACs) are not available at this time and thus will not be discussed in detail. Information regarding other modalities for management of VTE including thrombolytic agents and mechanical thrombolysis will be discussed elsewhere.

Heparins, including UFH and LMWHs, are a mainstay of initial VTE management in pediatric patients. UFH is often the first-line therapy in hospitalized pediatric patients who develop VTE and is used for primary prophylaxis of VTE in specific clinical settings including in individuals with congenital heart disease undergoing certain procedures or surgical interventions. UFH binds to antithrombin (AT), an endogenous anticoagulant, to induce a conformational change that makes AT a rapid inactivator of coagulation factors especially thrombin (Factor IIa) and Factor Xa. Binding of AT by heparin enhances the activity of AT on the order of 1,000- to 4,000-fold. In children, factors such as reduced levels of AT and prothrombin, reduced capacity to generate thrombin, and alterations in plasma binding may affect the action of UFH as compared to older individuals (5, 6).

Low molecular weight heparins are fragments of heparin with specific activity against activated factor X and less activity versus thrombin. Although the efficacy of LMWHs has not been proven in rigorous trials, they are used widely in pediatrics. Advantages of LMWH over UFH include a greater and more predictable bioavailability due in part to dose-independent clearance, longer duration of anticoagulation effect allowing for less frequent administration, and less frequent need for monitoring, which is importance in pediatric patients who may have poor venous access, and reduced complication rates of heparin-induced thrombocytopenia (HIT) and osteoporosis. LMWHs include enoxaparin (Lovenox^®^) and dalteparin (Fragmin^®^), noting that most clinical data available are a pediatric cohort with enoxaparin. Disadvantages of these LMWHs include twice daily subcutaneous injections, which can be problematic for some pediatric patients.

Fondaparinux (Arixtra^®^) is another anticoagulant utilized in pediatric VTE and is a synthetic pentasaccharide that causes an AT-mediated selective inhibition of factor Xa; unlike the LMWHs, fondaparinux has nearly pure anti-factor Xa activity. The advantages of fondaparinux over UFH are similar to those of the LMWHs; however, fondaparinux has some advantages over LMWH including once-daily dosing and no risk for neither HIT nor osteoporosis.

Another class of anticoagulants utilized in pediatric VTE, albeit infrequently, includes the DTIs. DTIs are short-acting agents that are more targeted than heparin and include the hirudin-like molecule bivalirudin, and small-molecule inhibitor argatroban, which are administered by continuous intravenous infusion. These molecules electively bind to and inhibit thrombin in both circulating and clot-bound forms. As compared to UFH, the pharmacokinetics are more predictable and these agents are not dependent on AT levels, which are physiologically low in children <6 months of age. These medications are used primarily in the setting of HIT, a rare but potentially life threatening condition mediated by IgG autoantibodies directed against platelet factor 4 in complex with heparin that occurs after heparin exposure.

Oral agents utilized in pediatrics patients for VTE thus far are limited to the vitamin K antagonist (VKA) warfarin (Coumadin^®^) in the United States. Warfarin works to inhibit the synthesis of vitamin K-dependent coagulant proteins, which include factors II, VII, IX, and X. Warfarin use in pediatrics is problematic in that it requires frequent monitoring, has numerous drug interactions, is affected by dietary intake of Vitamin K, and has a narrow therapeutic range. Additionally, warfarin is only available as a tablet and cannot be compounded into a liquid formulation making administration in young children difficult. VKAs in neonates are especially problematic due to the physiologically low levels of vitamin K-dependent clotting factors and the overall low vitamin K content of breast milk. VKA use in older children in contrast is often feasible with caregivers and physicians needing to balance the burden of frequent laboratory monitoring with dose adjustments with a VKA with the need for subcutaneous injections utilizing LMWHs.

Direct oral anticoagulants such as the factor Xa inhibitors [rivaroxaban (Xarelto^®^), apixaban (Eliquis^®^), edoxaban (Savaysa^®^)] and DTI [dabigatran (Pradaxa^®^)], which have been approved in adult patients for both treatment and prevention of VTE, neither have FDA-approved indications nor dosing in children that has yet been established. Use of these agents for children are appealing given that they are thought to require no specific monitoring and overall have a risk profile in adult studies that is equal to, or less than, VKAs. That said, extrapolation of adult data to pediatric patients at this time is premature and will not be addressed in this article. Fortunately, there are several clinical trials in pediatric patients addressing dosing, adverse events, and ultimately comparative efficacy versus standard anticoagulation of DOCAs that will ultimately guide their use in pediatrics.^1^^,^^2^^,^^3^

To date, comparative efficacy of various anticoagulants in pediatric patients has largely not been studied. The REVIVE trial was the first multicenter, international RTC to attempt to study comparative efficacy of anticoagulants (7). The study was an open-labeled RCT of LMWH compared to heparin and coumadin for the treatment of VTE in children that aimed to study the rates of recurrent VTE and death due to VTE during a 3-month treatment period. Unfortunately, the study was closed due to poor patient enrollment. Ongoing studies of the DOACs compared to anticoagulation currently used in pediatric patients will provide much needed data to guide hematologists on the specific safety and efficacy of these agents.

## Dosing and Monitoring

Dosing and monitoring guidelines in pediatrics have been established, often through extrapolation from adult data (Table 1). For UFH, no pediatric outcome-specific studies have established a pediatric range for UFH; therefore, therapeutic ranges have been generalized from adult VTE studies. Commonly, goals for therapeutic anticoagulation target an anti-Xa for UFH of 0.35–0.7 U/mL, thought to reflect to an activated partial thromboplastin time (aPTT) that correlates with a protamine titration of 0.2–0.4 U/mL (for purposes of this article an anti-Xa level of 0.35–0.7 will be assumed to reflect a aPTT of 60–85 s while acknowledging that this assumption may not hold true in pediatrics samples and will vary based on anti-Xa kit). Typically, initial UFH dosing involves a loading dose of 50–100 U/kg given intravenously over 10 min; there are little data to support this practice in pediatrics, especially in the neonatal population. Following initial bolus dose, maintenance dosing of 28 U/kg/h for infants (<1 year of age) and 20 U/kg/h for children ≥1 year is utilized. Choice of monitoring of anti-Xa for UFH versus aPTT in children is not well established and differs by institution. After initiation of UFH, anti-Xa/aPTT is then monitored 4–6 h post bolus and every 4–6 h after a dose adjustment (Table 1) (2). In patients who have difficulty achieving a therapeutic aPTT, checking AT levels is recommended, given AT supplementation may be required if sufficiently low.

**Table 1:** **Dosing and adjustment of anticoagulants**.

Class of medication/drug	Initial dosing	Subsequent dosing	Goal	Dose adjustment
UFH	50–100 U/kg IV (loading dose)	28 U/kg/h (age <1 year) 25 U/kg/h (age ≥1 year)	anti-Xa level 0.35–0.7/aPTT 60–85 s	aPTT <50 s; bolus 50 U/kg, increase by 10% aPTT <50–59 s; increase by 10% aPTT <60–85 s; no change aPTT <86–95 s; decrease by 10% aPTT <96–120 s; hold dose × 30 min, decrease by 10% aPTT <120 s; hold dose × 60 min, decrease by 15%

LMWH				
Enoxaparin	*Treatment dosing* 2 mg/kg/dose SC q 12 h (preterm neonates) 1.7 mg/kg/dose SC q 12 (term neonates) 1.5 mg/kg/dose SC q 12 h (age <2 months) 1 mg/kg/dose SC q 12 (age ≥2 months)		anti-Xa level 0.5–1 (treatment)	anti-Xa <0.35; increase dose by 25% anti-Xa 0.35–0.49; increase dose by 10% anti-Xa 0.5–1; no change anti-Xa 1.1–1.5; decrease dose by 20% anti-Xa 1.6–2; hold for 3 h and decrease dose by 30% anti-Xa >2; hold until anti-Xa 0.5–1 then decrease dose by 40%
	*Prophylactic dosing* 0.75 mg/kg/dose SC q 12 h (age <2 months) 0.5 mg/kg/dose SC q 12 h (age <2 months)		anti-Xa level 0.1–0.5 (prophylaxis)	anti–Xa <0.1; increase dose by 25% anti-Xa 0.1–0.5; no change anti-Xa 0.51–1; decrease dose by 20% anti–Xa >1; decrease dose by 30%
Dalteparin	129 ± 43 U/kg/dose SC q 24 h			

Fondaparinux	*Treatment dosing*			
	0.1 mg/kg SC q 24 h		anti-Xa level 0.5–1 (treatment)	anti-Xa <0.3; increase dose by 0.03 mg/kg anti-Xa 0.3–0.49; increase dose by 0.01 mg/kg anti-Xa 0.5–1; no change anti-Xa 1.1–1.2; decrease dose by 0.01 mg/kg anti-Xa >1.2; decrease dose by 0.03 mg/kg
	*Prophylactic dosing*			
	0.05 mg/kg SC q 24 h		anti-Xa level 0.1–0.5 (prophylaxis)	anti-Xa <0.1; increase dose by 25% anti-Xa 0.1–0.5; no change anti-Xa 0.51–1; decrease dose by 20% anti-Xa >1; decrease dose by 30%

VKA				
Warfarin	0.1–0.2 mg/kg (max dose 5 mg) PO q 24 h		INR 2–3	*Day 2–4 consider dose adjustment in day 2–4* INR 1.1–1.3; repeat initial dosing INR 1.4–3.0; give 50% of loading dose INR 3.1–3.5; give by 25% of loading dose INR >3.5; hold until INR <3.5 then restart at 50% dosing *Maintenance* INR 1.1–1.4; increase by 20% INR 1.5–1.9; increase by 10% INR 2.0–3.0; no change INR 3.1–3.5; decrease by 10% INR >3.5; hold until INR <3.5; restart at 20% decreased dosing

DTI				
Argatroban	0.75 μg/kg/min IV 0.2 μg/kg/min IV (hepatic impairment)		aPTT 1.5–2.5× baseline	
Bivalirudin	0.125–0.25 mg/kg (loading dose) IV	0.125–0.2 mg/kg/h	aPTT 1.5–3× baseline	

*UFH, unfractionated heparin; LMWH, low molecular weight heparin; VKA, vitamin K antagonist; DTI, direct thrombin inhibitor; IV, intravenously; SC, subcutaneously; PO, orally; aPTT, activated partial thromboplastin time; INR, international normalized ratio; anti-Xa, anti-Factor Xa level*.

Similar to UFH, therapeutic ranges for LMWH are largely extrapolated from adult VTE trials and are based on measurement of anti-Xa levels. For therapeutic dosing of LMWH, an anti-Xa of 0.5–1.0 U/mL from a sample obtained 4–6 h after a dose is considered in goal range; the initial anti-Xa level should be checked after the second or third dose is initiated. For patients receiving prophylactic anticoagulation to prevent recurrent VTE, an anti-Xa of 0.1–0.5 U/mL is typically considered goal. Dosing of LMWH varies by age with infants requiring often 50% increased dosing as compared to older children. Dosing for specific anticoagulants is listed in Table 1. Enoxaparin is the most widely utilized of the LMWHs in pediatrics and is typically initiated 2 mg/kg/dose every 12 h in preterm neonates, in 1.7 mg/kg/dose every 12 h in term neonates, 1.5 mg/kg/dose every 12 h for age <2 months, and 1 mg/kg/dose every 12 h for age ≥2 months (8, 9). Although enoxaparin has less activity against thrombin, in patients who have difficulty achieving a therapeutic anti-Xa level, checking AT levels to ensure that there is no significant AT deficiency should be considered. Fondaparinux is monitored using anti-Xa levels in a similar fashion to LMWHs and is initiated at a dose of 0.1 mg/kg/dose once daily.

Direct thrombin inhibitor use in pediatrics is largely in the setting of suspected or confirmed HIT. Anticoagulation goals have not been well established in this population. Argatroban manufacturer’s dosing guidelines include pediatric usage noting that in critically ill pediatric patients dosing is typically started lower than in adult patients. In general, therapy with argatroban is monitored utilizing aPTT with initial monitoring preformed within 1–3 h from medication initiation in patients without hepatic impairment and approximately 2–4 h after a dose change. Goal aPTT is typically 1.5–3 times baseline value and avoidance of aPTT > 100 s. With argatroban, dosing is typically a continuous infusion of 0.75 and 0.2 μg/kg/min in those with hepatic impairment without a bolus.^4^ As with argatroban, there is no established dosing range for bivalirudin in infants and children. The UtilizatioN of Bivalirudin on Clots in Kids (UNBLOCK) study found initial bolus dosing of 0.125 mg/kg followed by an initial infusion of 0.125 mg/kg/h of bivalirudin demonstrated efficacy and reassuring safety in a cohort of pediatric patients with acute VTE (10). Historically, monitoring of bivalirudin utilizing aPTT with a goal of 1.5–2.5× the baseline aPTT has been suggested; however, the UNBLOCK study demonstrated poor correlation between aPTT and plasma bivalirudin concentration suggesting limited utility of aPTT monitoring with this drug.

As with adult patients utilizing VKAs, target international normalized ratios (INRs) for anticoagulation are typically 2.0–3.0; to date, there have been no clinical trials to address optimal INRs for a pediatric cohort. In patients requiring anticoagulation for mechanical heart valves, a target INR of 2.5–3.5 mimic target INRs was fixed for adult patients. Warfarin is begun at a dose of 0.1 mg/kg, or alternatively with a loading dose of 0.2 mg/kg (maximum dose of 5 mg), with daily monitoring and dose adjustment in the first 5 days (Table 1) followed by monitoring of maintenance dosing (2). During the first 5 days of VKA use, and until the INR is at least 2.0 for two consecutive days, a heparin product should be utilized. This “bridging anticoagulant” is used to prevent against warfarin-induced skin necrosis, which occurs due to a relative decrease of vitamin K-dependent endogenous anticoagulants prior to a decrease of endogenous procoagulants. Repeat INR testing needs to be considered with changes to concomitant medication use or medical illness.

## Duration of Therapy

Duration of therapy for VTE in pediatric patients has been less well defined and is largely extrapolated from adult data. A recommendation of a 3-month course of anticoagulation for pediatric patients with provoked VTE has been based on the results of clinical trials in adults with a shorter, 6-week course, of anticoagulation considered in certain pediatric patient populations; these recommendations have not been based on evidence from pediatric trials. The American College of Chest Physicians Evidence-Based Clinical Practice Guidelines provides guidance for duration of therapy for pediatric patients with VTE in various settings, including consideration of longer duration of therapy in the setting of serious, unprovoked thrombosis and is largely used in pediatric practice to guide duration therapy (2). Notably, the pediatric recommendations from the CHEST Guidelines are largely based on expert opinion, case series, and relatively small studies rather than large RTCs, which have guided treatment recommendations in adult population.

## Adverse Events

With the use of any anticoagulant, the primary adverse event of therapy is bleeding. Given the lack of comparative efficacy studies in pediatrics, no adequately powered study has compared bleeding rates of one drug compared to another for treatment of UFH-related bleeding, given the short half-life of the medication, discontinuation of the medication is often sufficient. In the event of more severe bleeding or when immediate reversal may be needed preoperatively, use of protamine sulfate will rapidly neutralize UFH; guidelines for reversal are available (Table 2) (2). Although limited data are available for LMWH-related bleeding in the setting of overdosage, use of protamine can be considered (Table 2).^5^^,^^6^ It is important to note that the anti-Xa activity is never completely neutralized; with enoxaparin a maximum of 60% and dalteparin a maximum of 60–75%, of the anti-Xa activity is neutralized.

**Table 2:** **Anticoagulant reversal options for bleeding patients and over dosages**.

Class of medication/drug	Reversal strategy	Time since last dose of anticoagulant medication	Dosage of reversal agent
Unfractionated heparin	Protamine	<30 min 30–60 min 60–120 min >120 min	1 mg protamine per 100 U of heparin 0.5–0.75 mg protamine per 100 U of heparin 0.375–0.5 mg protamine per 100 U of heparin 0.25–0.375 mg protamine per 100 U of heparin

Low molecular weight heparin			
Enoxaparin	Protamine	≤8 h >8 h	1 mg protamine per 1 mg enoxaparin 0.5 mg protamine per 1 mg enoxaparin
Dalteparin	Protamine	N/A If bleeding 2–4 h after first protamine dose	1 mg protamine per 100 U of dalteparin 0.5 mg protamine per 100 U of dalteparin

Vitamin K antagonist (VKA)			
Warfarin	Vitamin K	N/A; if international normalized ratios >10 and no bleeding	
	Four-factor prothrombin complex concentrate	N/A; if VKA-associated major bleeding occurs	

Adverse events such as HIT are relatively rare in the pediatric population; however, this poses a potentially life threatening complication and narrows options for VTE treatment. Rates of HIT in pediatrics range from 0 to 3.7% (11). In adult patients, the pretest clinical scoring system commonly used known as the 4T score is not applicable in pediatric patients given the overall rarity of HIT in children versus adults.

For those individuals who require long-term preventative anticoagulation, other considerations need to be made such as the risk of osteoporosis with heparins. In adult patients, exposure to LMWH beyond 3–6 months may adversely affect bone mineral density; large epidemiologic studies of osteoporosis in pediatric patients with long-term heparin/LMWH exposure have not been conducted, but given the relationship between heparin use and osteoporosis in adults, this should likely be avoided in pediatric patients as well (12, 13).

## Conclusion

Given the increasing incidence of VTE in pediatric patients, it is crucial to understand treatment options for VTE including ways in which the hemostatic system and anticoagulant dosing and monitoring are different in this cohort as compared to adult patients. Notably, there is a lack of robust research aimed at addressing dosing, monitoring, safety, comparative efficacy, and duration of therapy to guide optimal care in pediatric patients, which offers areas for research focus for the future.

## Author Contributions

LM and GY conceptualized and researched the article and participated in manuscript preparation.

## Conflict of Interest Statement

GY participates in the Kids-DOTT trial discussed in the “Goals of Treatment” section of this review article. The other author declares that the research was conducted in the absence of any commercial or financial relationships that could be construed as a potential conflict of interest.

## Footnotes

^1^Bayer. *EINSTEIN Junior Phase III: Oral Rivaroxaban in Children with Venous Thrombosis (EINSTEIN Jr)*. Available from: www.clinicaltrials.gov.

^2^Pfizer. *Apixaban for the Acute Treatment of Venous Thromboembolism in Children*. Available from: www.clinicaltrials.gov.

^3^Boehringer Ingelheim. *Open Label Study Comparing Efficacy and Safety of Dabigatran Etexilate to Standard of Care in Paediatric Patients with Venous Thromboembolism (VTE)*. Available from: www.clinicaltrials.gov.

^4^Argatroban. *GlaxoSmithKline*. Houston, TX. Available from: http://www.accessdata.fda.gov/drugsatfda_docs/label/2008/020883s014lbl.pdf.

^5^Lovenox. *Sanofi-Aventis*. Bridgewater, NJ. Available from: http://products.sanofi.us/lovenox/lovenox.html#section-12.

^6^Dalteparin. *Pfizer*. New York, NY. Available from: http://www.accessdata.fda.gov/drugsatfda_docs/label/2015/020287s062lbl.pdf.
